# Trust in Government Actions During the COVID-19 Crisis

**DOI:** 10.1007/s11205-021-02772-x

**Published:** 2021-08-24

**Authors:** Marc Oliver Rieger, Mei Wang

**Affiliations:** 1 University of Trier, Research Cluster “Cultures in Transitions”, 54296 Trier, Germany; 2 WHU – Otto Beisheim School of Management, Vallendar, Germany

**Keywords:** SARS-Cov2 pandemics, Government trust, Perception of government interventions, Stringency, Lock-down, Media freedom, Conspiracy theories, H12, I18

## Abstract

The worldwide COVID-19 pandemic puts countries and their governments in an unprecedented situation. Strong countermeasures have been implemented in most places, but how much do people trust their governments in handling this crisis? Using data from a worldwide survey, conducted between March 20th and April 22nd, 2020, with more than 100,000 participants, we study people’s perceptions of government reactions in 57 countries. We find that media freedom reduces government trust directly as well as indirectly via a more negative assessment of government reactions as either insufficient or too strict. Higher level of education is associated with higher government trust and lower tendency to judge government reactions as too extreme. We also find different predictors of perceived insufficient reactions vs. too-extreme reactions. In particular, number of COVID-19 deaths significantly predicts perceived insufficient reactions but is not related to perceived too-extreme reactions. Further survey evidence suggests that conspiracy theory believers tend to perceive government countermeasures as too strict.

## Introduction

Pandemics are nothing new to humankind, but in our globally interconnected world, an infectious disease can spread at a breathtaking speed. This was the case with the coronavirus disease 2019 (COVID-19) caused by the SARS-Cov2 virus. After it was first detected in Wuhan, a city from China, it soon started spreading around the globe, forcing most countries to introduce strict countermeasures to curtail the spread of the disease and to avoid a failure of their healthcare systems. In many instances “lock-downs” were implemented for several weeks, reducing social and economic activity to the minimum amount possible. The effects, for example, on mobility and on business, are well documented (Rieger and Wang 2020; Atkeson 2020; McKibbin and Fernando 2020).

In this article, we want to take a look at how people perceive the reactions of their governments, and whether they see these reactive countermeasures as “insufficient” or “too strict.” Reactions varied a lot (Hale et al. 2020), so it is natural to wonder how people perceived these actions and what were the most important factors contributing to their trust in governments. In a case study of Great Britain, Newton (2020) suggests that the inaction and misinformation by the British government caused public mood to swing from trust to distrust in their government. Consistent with this observation, our cross-country empirical analysis shows that trust in government is more correlated with perceived lack of government responsiveness as compared to the perceived too-extreme countermeasures.

Furthermore, we study how different factors such as education, media freedom, actual stringency level and death rate, affect the judgment of government policy responsiveness. Our results imply that the evaluation of insufficient or too extreme responses seem to be influenced by different factors. For example, higher COVID-19 fatality rate predicts perceived insufficient government response, whereas it turns out to be unrelated to whether the policy responses were perceived as too strict or not. We conducted our own survey in Germany to investigate how conspiracy believers evaluate government reactions. Our findings show that conspiracy theory believers tend to think that the government reaction is too strict, although such judgment is less important in predicting trust in government as compared to the perception of insufficient reaction.

Our paper is structured as follows: in Sect. 2, we review the relevant literature. In Sect. 3, we present the methodology, data sources, and variable structures. Section 4 describes the results, particularly regarding the heterogeneity of government trust, and the explanatory factors for trust and for assessment of government actions. This is followed by conclusions in Sect. 5.

## Literature Review and Hypotheses Development

### Trust as Evaluation on Policy Responsiveness

Political trust has many possible layers and definitions (Norris 2017). We follow a rationalist evaluative framework, where trust implies that “subject A trust objects B to do x” (van der Meer 2017). In the context of our study, we investigate to what extent *the people* (A) trust *the government* (B) to *manage the Covid-19 pandemics and take care of its citizens* (x). Political trust is therefore presumably based on the assessment of performance of political institutions, and it depends on to what extent the citizens perceive that the governance has produced or can produce desired outcomes (North 1990).

Most empirical studies focus on macroeconomic performance (van der Meer 2017), while paying less attention on other domains (Kumlin and Haugsgjerd 2017). The global Covid-19 pandemic provides a unique chance to observe government reactions and political trust during the crisis. In fact, some authors argue that poor performance and unresponsiveness of political institutions during the crisis are even more important causes of the decreasing levels of trust than the economic crisis itself (Torcal 2014; Ervasti et al. 2019; Denters et al. 2007). With regard of government reaction, the citizens can always be unhappy if they think governments has interfered too much or too little. This applies to Covid-19 as well. Citizens can be unsatisfied with the lack of countermeasures to stop the spread of the virus, but they may also complain the stringency policies to be too strict, which may constrain freedom and hurt certain economic sectors. Therefore, the governments around the world are facing difficult trade-offs regarding how to respond to this health crisis in appropriate ways. In this study, we investigate how the perceived responsiveness affects the trust in government, and what are the underlying factors of the assessment on government responsiveness.

Table 1 summarises the basic structure and hypotheses that guide our data analysis. We expect government trust is based on the evaluation of the policy responses during COVID-19. In the best case, the citizens are satisfied and consider the government reactions as appropriate. Otherwise, they may consider the government response as either insufficient or too extreme, both of which would reduce the trust in government handling the crisis, as indicated in the top right columns in Table 1. In the following subsections, we discuss how characteristics of trusters (citizens) and trustees (governments) will be related to assessment on policy responsiveness and trust in government.

### Trusters: Education, Media Environment, Beliefs

In the evaluative framework, citizens are the trusters who estimate the trustworthiness of the government by evaluating its performance. The subjective assessment of policy responsiveness tends to be heterogenous across individuals. It depends on the ability and motivations to search and process relevant information to form judgment. Education and media are two important sources of information.

Empirical studies show that the relationship between education and political trust is complicated and context-dependent (Mayne and Hakhverdian 2017). Education serves as an important component of early socialisation that helps to form political opinions later in life. Most school education presents an idealised picture of institutional foundations, thus helps to cultivate social norms of trusting the political system. In fact, many studies document a positive relationship between trust and education, e.g., Anderson and Singer (2008), Hetherington (1998).

Education, however, may also reduce the level of trust, as documented in some studies (Cook and Gronke 2005; Chang and han Chu 2006). The potential reason is that people with more education tend to have higher expectations of government performance, and consequently they are more critical and less satisfied. Furthermore, the relationship between education and trust depends on the situational factors. Generally, we believe that education improves cognitive skills to acquire and process information, thus forming more accurate and rational beliefs on government performance. For example, Hakhverdian and Mayne (2012) find that higher-educated citizens are more sensitive to the corruptions—the relationship between education and trust is negative in more-corrupted countries, but positive in clean societies. Besides these causal mechanisms, some scholars argue education can simply be a proxy of social status or cognitive ability, and education *per se* may not exert additional impacts on trust (Mayne and Hakhverdian 2017).

If education can be considered as a proxy of cognitive skills and information processing ability, then in the context of Covid-19 pandemics, more educated people should be more knowledgable and more informed about the importance of countermeasures in curtailing the spread of pandemics. Therefore, we hypothesise that people with higher education are less likely to criticise the government reaction to be too strict, and more likely to see the reaction as no sufficient. Given the above literature on ambiguous relationship between education and political trust, we do not have a straightforward hypothesis on the direct relationship between education level and trust in government (see Table 1).

Media is important information source beyond close social relationships. Many people collect information from various media to judge the government performance. While higher media freedom increases political knowledge and participation (Leeson 2008), it is also commonly believed that the frequent negative news in the media tend to erode public trust in government and political figures (Newton 2017). Using the data from the 2008 World Public Opinion poll, Yakovlev and Gilson (2015) find that press freedom increases the trust in foreign leaders as compared to domestic leaders. They also point out that only leaders in the three countries with the lowest press and political freedom (China, Iran, and Russia) received consistently higher trust at home than abroad. Based on these studies, we expect more media freedom will be associated with more negative evaluation of government policies, i.e., with increased chance to perceive the policy reaction as either too lax or too strict. Consequently, media freedom is likely to reduce government trust. Free media, however, may also increase government trust because more transparent information is a good foundation for mutual trust. Therefore, similar to education, the direct influence of media to government trust is ambiguous (see Table 1).

We have conducted additional surveys on conspiracy beliefs. The news on COVID-19 from various media sources range from high-quality scientific information to all kinds of conspiracy theories, e.g., that it had been developed as bioweapon, that it is in reality caused by 5G cell phone radiation, or that it is nothing more than a made-up disease. The fact that conspiracy believers tend to distrust science and have no problem believing in contradicting conspiracy stories suggests the existence of a conspiracy mentality (Wood et al. 2012; Imhoff and Lamberty 2020; Bruder et al. 2013; Goertzel 1994). Previous studies have shown beliefs in conspiracy theories correlate with distrust in governments, even when the government services and institutions are clearly not connected to the allegations stated in conspiracy theories (Einstein and Glick 2015; Kim and Cao 2016). Researchers also show that conspiracy beliefs influence containment behaviour during COVID-19 (Imhoff and Lamberty 2020; Karić and Medjedović 2021; Rieger 2020): the more respondents believe in conspiracy theories, the less they like to engage in transmission reducing activities, e.g., social distancing or wearing masks. Karić and Medjedović (2021) show that one channel for this effect is reduced political trust. Based on these studies, we expect that conspiracy believers are more likely to perceive policy reactions as too strict.

### Trustees: Government Quality, Objective Policy Responses, Policy Outcomes

In our evaluative framework of trust, the governments are the trustees, whose performance would be evaluated by citizens. Assuming a rational Bayesian updating process as our benchmark, people would revise their prior beliefs by incorporating new information.

The prior belief can be formed based on the past performance, proxied by government effectiveness in our analysis. It is a measure of government quality and competence based on the track record. We expect that more effective governments will win more political trust from their citizens. We also expect that the policy responses by the more-effective governments are more likely to be seen as too extreme rather than insufficient (see Table 1, the last fourth row).

To update beliefs about how competent the government can handle the ongoing crisis, one can observe the policy reactions and the outcomes. In reality, it is easily said than done. In the domain of macroeconomic policies, for example, even the most brilliant economists like Hayes and Keyes disagreed about the optimal policy responses to an economic crisis. Studies have revealed that policy performance, especially economic performance, stimulates political trust in well-established democracies (Anderson 2009; Clarke et al. 1993), although some studies show no effect of economic performance (Hakhverdian and Mayne 2012).

Health experts, however, have reached far higher consensus on how to deal with a pandemic. They developed well-calibrated models for predicting the impacts of infection reduction by various countermeasures, such as mask wearing, social distance, and quarantine. The causal mechanisms between the stringency policies and infection rates are much less ambiguous as compared to the link between macroeconomic policy intervention and economic performance. Therefore, we explore to what extent the subjective evaluation of policies responses are associated with stringency policies and outcomes (e.g., Covid-19 deaths). Such information can be gained from various media sources and personal experience. We expect the current and average stringency measures to be positively related to perceived too-extreme reactions, and negatively related to the perceived insufficient reactions. Additionally we expect the policy outcomes, as proxied by COVID-19 death rates, to be positively associated with too-extreme policies, and negatively related to perceived insufficient policies. (see Table 1, the last three rows).

**Table 1:** Hypotheses on relationship between trust, perceived policy responsiveness and other variables

	*Perceived policy responsiveness:*		
	Too extreme	Insufficient	Government trust
*Perceived responsiveness*			
Too extreme			− (−*)
Insufficient			− (−*)
*Truster*			
Education	−(−*)	$+$ (/)	± ($+$*)
Media freedom	$+$ ($+$*)	+ ($+$*)	± (−*)
Conspiracy beliefs	$+$ ($+$*)	(/)	
*Trustee*			
Government effectiveness	$+$ ($+$*)	− (−*)	$+$ ($+$*)
Current stringency	$+$ (/)	− (−*)	
Average stringency	+ ($+$*)	− (/)	
COVID-19 deaths	− (/)	$+$ ($+$*)	

± designates cases in which both directions could be predicted by theory. The empirical relationship is presented in the bracket, where one star (*) represents a significant relationship at .05 level. Slash (/) represents no significant relationship

## Data and Methodology

### Government Trust and Perceived Government Reactions During COVID-19

Table 2 summarizes the main variables in our study. Detailed information about these variables can be found in the referenced papers.

**Table 2:** Descriptive statistics

Variable	Measurement	Mean	SD	*N*	Source
*Individual level*					
Government trust	1(= strongly distrust) to 5(= strongly trust)	2.98	1.5	101411	Fetzer et al. (2020a, 2020b)
Perceived insufficient reaction	1(= somewhat insufficient); 2(= not at all sufficient); otherwise 0	0.8	0.78	101411	Fetzer et al. (2020a, 2020b)
Perceived too-extreme reaction	1(= somewhat too extreme); 2(= much too extreme); otherwise 0	0.06	0.28	101411	Fetzer et al. (2020a, 2020b)
Perceived insufficient reaction in Germany	1 (= rather too slow or lax); 2(= far too slow or lax); otherwise 0	0.25	0.48	550	Surveys by the authors
Perceived too-extreme reaction in Germany	1(= rather too fast or restrictive); 2(= far too fast or restrictive); otherwise 0	0.12	0.37	550	Surveys by the authors
(Self-reported) Health	1(= poor) to 4(= excellent)	3.1	0.72	101411	Fetzer et al. (2020a, 2020b)
Conspiracy Total	Aggregate scores of beliefs in seven conspiracy theories.	10.9	3.6	455	Rieger and He-Ulbricht (2020)
Likelihood of voting AfD (right populist party)	1(= definitely not) to 6(= definitely yes)	1.39	1.06	124	Rieger and He-Ulbricht (2020)
Evaluation of government on left-right scale	− 4(= way too left) to $+$ 4(= way too right)	0.41	1.4	1626	Rieger and He-Ulbricht (2020)
Political interest	1(= very little) to 5(= very much)	3.22	0.99	929	New data set

		Mean	SD	Countries	Source
*Country level*					
Average Stringency since start	Stringency index from 0 to 100%	16.1	7.8	55	Hale et al. (2020)
Average Stringency since first death	Stringency index from 0 to 100%	71.1	16.6	55	Hale et al. (2020)
Press Freedom	Index, values among our countries from 7.84 to 76.68	24.3	12.9	55	Reporters without Borders (2020)
World Governance Index	Normalized index, values among our countries between -1.85 and +1.83	-0.38	1.08	55	World Bank

Our main analysis is based on data from the online survey by Fetzer et al. (2020b), covering more than 170 countries. This survey was initially advertised worldwide on Twitter and encompasses the time period from March 20th, 2020 to April 22nd, 2020. We only consider countries with at least 200 participants. This leaves data for 57 countries with $N=106,010$ participants, out of which 57% were female, the average age was 39.1 years, and 44% were married or living with a partner.

The subjective evaluations were elicited from the survey respondents around the world. They were asked to express their opinion regarding whether the governments were too fast or too slow in reacting to the pandemic, which is formulated as the following two questions:

- *Government trust:* How much do you trust your country’s government to take care of its citizens? (1 = Strongly distrust; 2 = Somewhat distrust; 3 = Neither trust nor distrust; 4 = Somewhat trust; 5 = Strongly trust)
- *Perceived reaction:* Do you think the reaction of your country’s government to the current coronavirus outbreak is appropriate, too extreme, or not sufficient? (The reaction is much too extreme / somewhat too extreme / appropriate / somewhat insufficient / not at all sufficient)

To facilitate interpretation and further analysis, we decomposed the answers to the *perceived reaction* question into two subindices, so that higher values always indicate stronger dissatisfaction with the government reaction (i.e., either too extreme or insufficient), similar to the decomposition methods in Fetzer et al. (2020a). The main reason is that the variable is not monotonic with regard to satisfaction. The decomposition make both variables monotonic, i.e., highest number corresponds to highest dissatisfaction either from perceived too-extreme or insufficient reactions.As we will see later from the results, these two types of judgment are determined by different underlying factors, especially regarding the predictive power of COVID-19 deaths. The two indices of perceived reaction are:

- *Perceived insufficient reaction:* The reaction is not at all sufficient (= 2); somewhat insufficient (= 1); otherwise (= 0).
- *Perceived too-extreme reaction:* The reaction is much too extreme (= 2); somewhat too extreme (= 1); otherwise (= 0).

As alternative measurements of trust and perceived reaction, we used the 2020 Democracy Perception Index (DeVeaux and Dölitzsch 2020). These data are based on representative samples from 53 countries around the world from which we could use 46 countries for our study. Since the precise dating of the individual-level data is unfortunately not available for these data, the analysis is only possible on the country level. Nevertheless, this provides us with a very useful comparison of the country-level trust.

The two items from the Democracy Perception Index survey that we used were:

1. *Country trust:* How well do you think your country is responding to the coronavirus (COVID-19) crisis? (Very poorly / somewhat poorly / somewhat well / very well)
2. *Perceived reaction:* Do you think your government is doing too much or not enough to restrict the movement of people in your country? (Too much / right amount / not enough)

### Policy Stringency and Death Rates

To measure the actual government policy reactions, we use data on policy *stringency* (Hale et al. 2020) that provides us with a numerical value describing the amount of restrictions in place in a given country for each day during the period under investigation. Since perceptions of government actions will depend not only on the current measures but also on the past actions, we calculate the current stringency index for each country on the day of the survey (*current stringency *) and the average stringency from the first confirmed death from COVID-19 in that country (*average stringency since first death*).

We use data from Dong et al. (2020) on numbers of COVID-19 cases and deaths on the country level for each country under study. For our statistical analysis, we focus on deaths, since the number of cases crucially depends on testing efforts and is consequently seen as less indicative of the extent of an outbreak when comparing different countries, whereas the numbers of deaths are usually considered to be more accurate. We use the data from the previous day to correct for a time lag in reporting.

### Media Freedom and Government Effectiveness

The variable *media freedom* is taken from World Press Freedom Index at the country-level (Reporters without Borders 2020). In addition, we control the *government effectiveness* on the macro-level using the subindex from the World Governance Index (World Bank n.d.).

### Individual-Level Variables

The variable *education* is measured by the number of years of education that the participant completed. We also control the self-reported overall *health* of the participants from the survey by Fetzer et al. (2020b). On a scale from 1 (poor) to 4 (excellent), they stated their health on average as 3.1. We also control other demographic variables, including gender, age, and marital status.

### Additional Online Surveys in Germany: Perceived Reactions and Conspiracy Theory Beliefs

To explore the relationship between beliefs and perceived government reactions, we conducted two online surveys in Germany on April 21–23, 2020 among 268 subjects and mainly on May 18–25, 2020 among 248 subjects^1^, both advertised at the University of Trier, a medium-sized German university, and conducted using Unipark.^2^ In the first survey, 64% of the participants were university students, 63% females, and the average age was 28 (from 18 to 77). In the second survey, 77% were university students, the average age was 26 (from 18 to 65, and 62% were females. Since all students and employees of the universities were invited to participate in the survey, the sample is diverse in age, gender and education, but is on average much younger than the overall German population. The gender is fairly balanced, albeit with a slight overweight on female. In both cases, one of the participants won a prize of 50 euros (as announced in the advertisement to incentivize participation). In these surveys, we asked essentially the same question as Fetzer et al. (2020b)^3^:

- *Perceived reaction:* All in all, how would you assess the reactions of German politicians to the coronavirus? (The reaction is far too slow or lax / rather too slow or lax / balanced / rather too fast or restrictive / far too fast or restrictive)

Similar to previous coding of perceived reactions from the survey by Fetzer et al. (2020b), we defined two variables: *perceived insufficient reaction in Germany* and *perceived too-extreme reaction in Germany*.

In addition, we elicited information about a tendency to be receptive for conspiracy theories regarding COVID-19. More specifically, we asked participants to state whether they agree with the following statements:

- The media want to hide information about the coronavirus from us.
- The hype about corona was only caused by pharmaceutical companies and other groups that benefit from it.
- The virus serves our politicians only as a pretext to undermine our basic rights.

We provided four answer options: do not agree / partially agree / mostly agree / fully agree.

We also directly elicited belief in some of the most popular conspiracy theories, namely:

- The US secret service developed the virus and brought it to Wuhan in order to specifically damage China.
- China developed the virus in a laboratory for bio-weapons, from where it spread by accident.
- Covid-19 is connected to the expansion of the 5G mobile phone network.
- Pharmaceutical companies in conjunction with Bill Gates started the infection in order to make money with a vaccine they had patented.

These items were mixed with statements that reflect the scientific consensus (at least at the time of the survey), e.g., that the virus spread from animals (bats or pangolins) to humans, originated in Wuhan (China) etc. All items could be judged on a five-point Likert scale (very unlikely; unlikely; average probability; probably; very likely).

We defined a composite score *Conspiracies Total* as the sum of the answers to all seven of the above conspiracy items, where scales were coded with the numbers 1 to 4 or 5, respectively. We considered subjects with a score of more than 10 (26.9% in the first, and 23.8% in the second survey) as “conspiracy tendency” subjects.

Regarding the conspiracy theories, we also asked participants whether they had ever heard about them before. Indeed, that was the case for between 43% and 70% of the subjects in the first, and between 51% and 75% in the second survey.

We measured the political positioning of the respondents in several survey waves from September 2020 to January 2021. In September 2020, we asked respondents to state for each of the parties in the German parliament: “How likely are you to vote for the following parties in the next federal election?” Answers were on a 6-point Likert scale (see Tab. 2 for details). Between October 2020 and January 2021, we asked how respondents would evaluate the current German government on a classical left-right spectrum between “− 4 = way too left” and “$+$ 4 = way too right”. The German government at that time was formed by the two traditional center-left/center-right parties in Germany, the SPD and the CDU/CSU. In this way, we could therefore indirectly deduce the political viewpoint of the respondents.

Differences between most parties regarding COVID-19 politics were, at least for a long time, rather small. There was, however, one noticeable exception; the AfD, a right-wing populist party that was highly vocal in its demands to reduce protection measures against the pandemic. The AfD (as well as several other European right-wing parties) was also attracted by conspiracy theories (Bieber 2020). We therefore use the likelihood to vote for the AfD as a variable in our analysis.

In a follow-up wave in February 2021, we also measured political interest with the following question: “How interested are you in politics in general?” This item was elicited on a 7-point Likert scale with answer options from “very little” to “very much”.

## Data Analysis and Results

### Government Trust, Perceived Reactions, and Stringency Measures

**Fig. 1:**
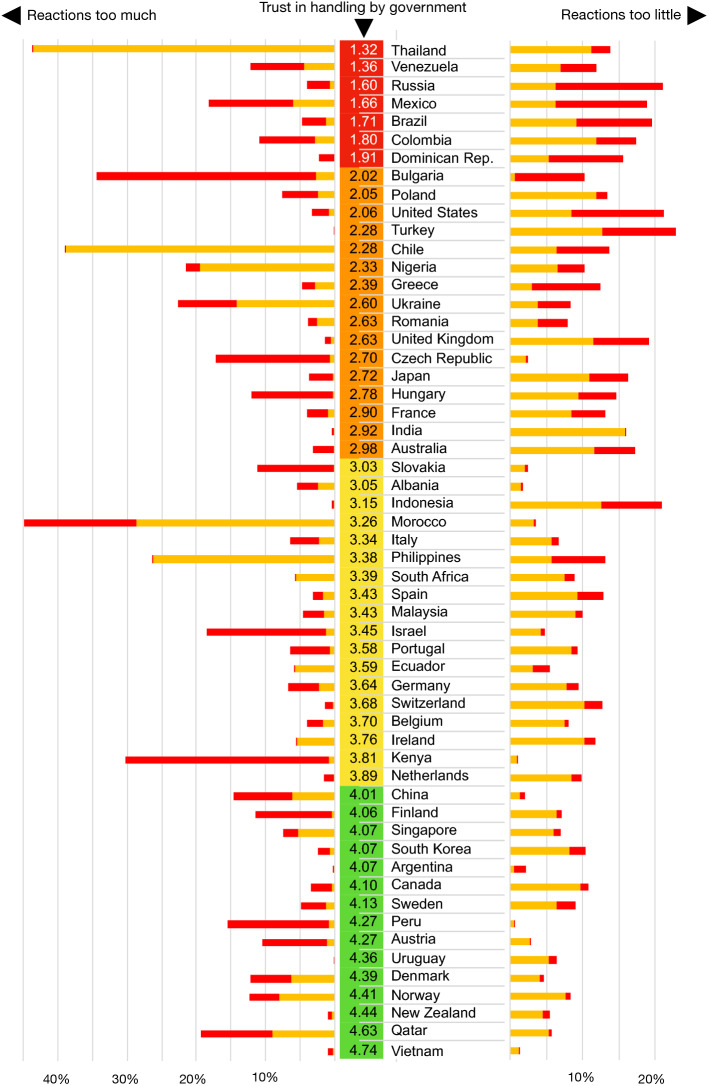
Perceptions of governments by participants from the respective countries, sorted by government trust (from 1 =“strongly distrust” to 5 =“strongly trust”) based on the survey by Fetzer et al. (2020b). Red = reaction much too extreme/not at all sufficient; orange = somewhat too extreme/somewhat insufficient. (Color figure online)

**Fig. 2:**
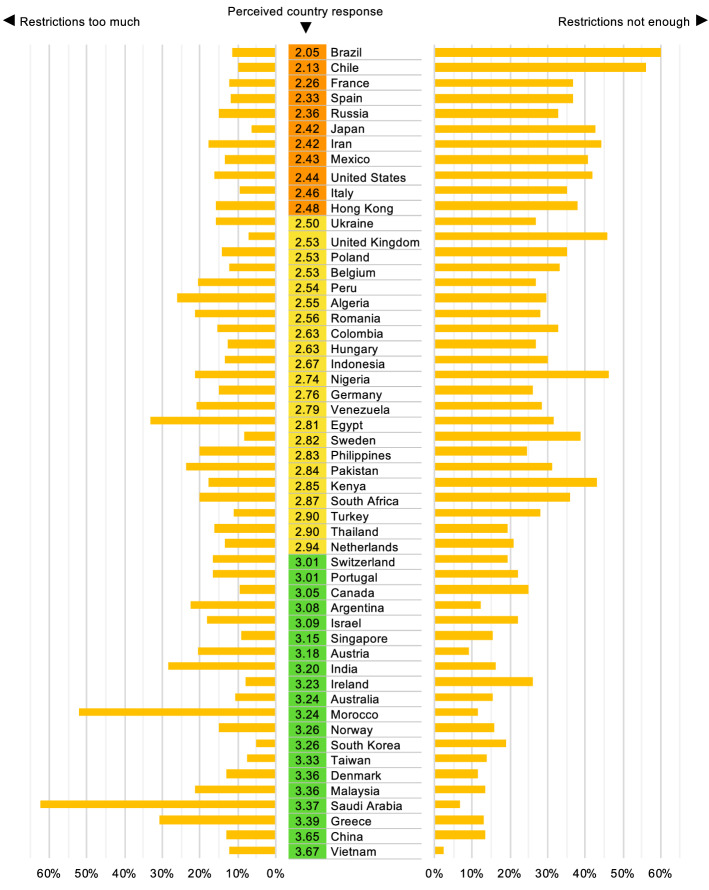
Perceptions of country reactions by participants from the respective countries (from 1 =“very poorly” to 4 =“very well”) and proportion of people considering the reactions too little or too much, based on the Democracy Perception Index survey by DeVeaux and Dölitzsch (2020)

Figure 1 displays the government trust from lowest to highest together with perceived reactions (too much vs. too little) based on the data of Fetzer et al. (2020b). Similarly, Fig. 2 shows the ranked country trust with the perceived reaction (too much vs. not enough) based on the 2020 Democracy Perception Index (DeVeaux and Dölitzsch 2020). Both figures reveal a large cross-country heterogeneity of the perceived government reactions around the world. This comes as no surprise in light of the news being full of discussions on countries with very harsh (e.g., China) or very relaxed (e.g., Sweden) policies in tackling the pandemic and when considering the reported differences in performances of governments worldwide in this situation. The Vietnamese government shows up as the most trusted government worldwide in both surveys. Given the extremely low number of infections and death rate in Vietnam despite its close connections with China, this is to some extent understandable. On the other end of the list, we find countries whose governments have been heavily criticised in the media for their handling of the situation, thus the measurement is in line with expectations.

Even though the wording of the questions is not the same in these two surveys, the patterns of both studies are similar. Indeed, the correlation between both datasets is fairly high: government trust (Fetzer et al. 2020b) and country trust from the Democracy Perception Index DeVeaux and Dölitzsch (2020) are correlated with .57 ($p<.001$). Moreover, the perceived insufficient reactions of the two datasets are correlated with .47 ($p<.001$), and the perceived too-extreme reactions with .40 ($p<.001$). We will not use the Democracy Perception Index survey for the further regression analysis because the precise dates are not available at the individual level. On the other hand, since the survey of the Democracy Perception Index has used representative samples, this gives us more confidence in the reliability of survey results from Fetzer et al. (2020b).

**Fig. 3:**
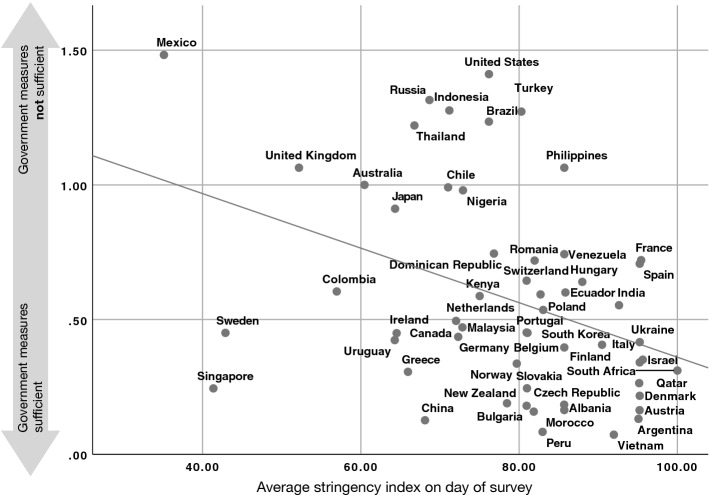
Average stringency on day of survey (x-axis) versus opinion of people in a country that the government measures are not sufficient (y-axis)

**Fig. 4:**
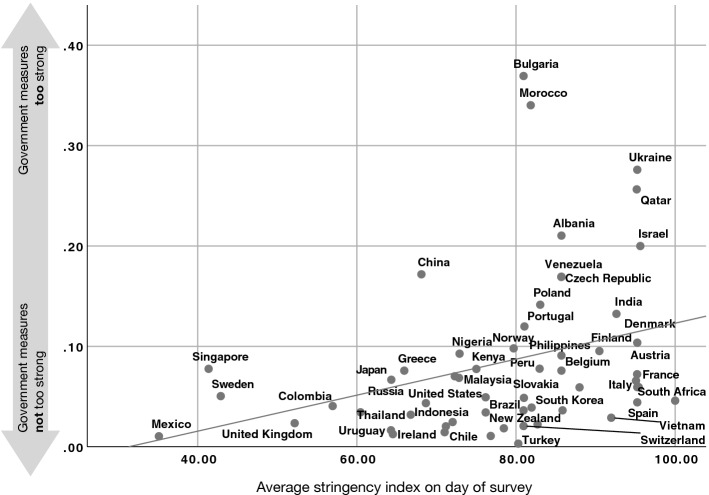
Average stringency on day of survey (x-axis) versus opinion of people in a country that the government measures are too extreme (y-axis)

Figures 3 and 4 demonstrate the relationship between actual stringency measures and perceived reactions in the 56 countries that we studied. As expected, with stronger stringency measures, people are less likely to perceive government reactions as insufficient ($r=-\,0.38$, $p=0.004$, Fig. 3), and more likely (not statistically significantly) to perceive the measures to be too extreme ($r=0.32$, $p=0.16$, Fig. 4).

### Determinants of Government Trust

A glance at Fig. 1 already suggests some relation between the degree of perceived reactions and the overall government trust. We now take a closer look at the factors leading to a high (or low) trust in the government in handling this crisis. All in all, we have a mix of variables on the individual level and on the country level, where some of the latter ones vary from day to day. In order to examine the factors more closely, we conduct multilevel model regressions. Moreover, we used robust regressions with clustered standard errors on country level, weighted to adjust the number of participants per country and the demographics of the participants (see Fetzer et al. 2020b for details). We also conducted additional robustness tests without weighting and outliers. For the outlier detection, we calculated Cook’s distance and dfbeta for the regressions of average stringency versus government measures on country level (compare Figs. 3, 4). Only for Mexico we found a Cook’s distance larger than 0.5, namely 10.9. The dfbeta was for all countries, but Mexico lower than 0.5, but for Mexico 4.7. Thus, we removed the data from Mexico for our robustness test.

We see that both perceived insufficient reaction and too-extreme reaction play a huge role for government trust (Table 3). In fact, if we use on OLS regression (not reported here), we can explain around 45% of the total variation just with these two variables. In other words, during the COVID-19 crisis, a government is judged to a large extent by the timing and appropriateness of its countermeasures.

The last two models in Table 3 show that media freedom reduces government trust. This implies that governments in countries that have highly censored media tend to be seen as *more* trustworthy. It seems, unfortunately, that censorship works to some extent to establish trust in governments. This is in line with the previous findings that show leaders in countries with lower press and media freedom (e.g., China, Iran, Russia) receive a higher level of domestic trust (Yakovlev and Gilson 2015). A priori, the result might also be induced by self-censorship: participants might not answer honestly in countries with low press freedom, since they might simply be afraid. Recent research, however, suggests that this is usually not the case (Calvo et al. 2019).

As expected, the “output measure” is a very strong predictor, i.e., the number of deaths reduces government trust. Stringency measures, on the other hand, tend to increase government trust, particularly if these have already been implemented in the early stages of the outbreak.^4^ Demographic factors (gender and age) play a certain role, as does health. The perception of insufficient reaction is by far the most important factor in all models.

**Table 3:** The perception of a too-weak response to the crisis is the strongest factor explaining the trust in the government, even when controlling for the effect of the crisis (number of deaths in the country at time of survey) and a number of other factors. Model 4 is the robustness test (without weighting and omitting the outlier country Mexico)

Government trust	Model 1	Model 2	Model 3	Model 4
Perceived insufficient	− 1.144***	− 1.158***	− 1.149***	− 0.904***
reaction	(− 17.73)	(− 11.43)	(− 11.00)	(− 184.16)
Perceived too extreme	− 0.558**	− 0.606***	− 0.579**	− 0.600***
reaction	(− 2.58)	(− 3.28)	(− 2.82)	(− 49.10)
Media freedom			− 0.026***	− 0.012*
			(− 4.86)	(− 2.05)
Government effectiveness			− 0.020	0.008
			(− 0.36)	(0.09)
Current stringency			− 0.004	0.000
			(− 0.88)	(0.56)
Average stringency since			0.040***	0.005**
the first death			(3.93)	(2.73)
COVID-19 deaths			− 0.000***	− 0.000**
(in thousands)			(− 4.19)	(− 2.70)
Education		0.008	0.008	0.004***
		(0.54)	(0.51)	(4.95)
Age		0.01**	0.009*	0.003***
		(3.01)	(2.27)	(12.13)
Female		− 0.292*	− 0.321*	− 0.005
		(− 2.28)	(− 2.26)	(− 0.74)
Health (self-assessed)		0.203***	0.221**	0.106***
		(3.32)	(2.56)	(23.51)
Married		0.043	0.016	− 0.049***
		(0.32)	(0.11)	(− 7.10)
Constant	3.865***	3.195***	3.633***	3.568***
	(35.8)	(9.35)	(6.02)	(19.25)
*N*	105057	105057	100605	97605
Countries	57	57	55	54
Wald $\chi ^2$	333.99	332.08	635.69	34166
Log pseudolikelihood	− 749.17	− 733.28	− 636.43	− 138599

*, **, *** correspond to significance on the 5%, 1%, and 0.1% level

### Structural Equation Model: Perceived Reactions as Mediators

**Fig. 5:**
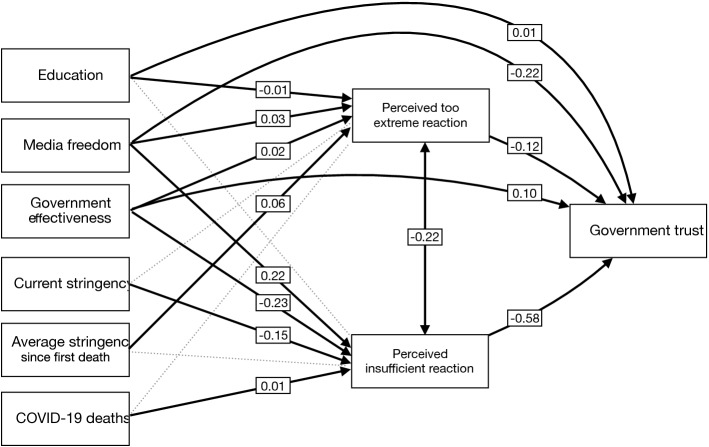
Structure equation model. Values represent standardized estimates. Significant paths ($p<.05$) are represented in solid lines

As discussed in the literature review, evaluation on policy responsiveness can be influenced by micro-and macro-level factors, such as education, media environment, and governance effectiveness. Hence, we test a structural equation model, taking the endogeneity issue into consideration. The model fit the data very well ($\chi ^2=81605$, $df=21$, $p<.001$; CFI = .998; TLI = 0.984; RMSEA = .025). As shown in Fig. 5, we observe a partial mediation effect of perceived reactions, i.e., education, media freedom and government effectiveness affect government trust directly and indirectly via perceived reactions. Consistent with the previous regression results, the model also shows that perceived insufficient reaction is a much stronger predictor of government trust than the perceived too-extreme reaction. Moreover, perceived too-extreme reaction is not related to COVID-19 deaths, whereas perceived insufficient reaction is significantly related to a higher death rate. It is interesting to see that media freedom reduces government trust directly and indirectly through reduced satisfaction with government responses.

### Conspiracy Theory Beliefs and Perceived Government Reactions: Further Evidence from Germany

It is intriguing to see from our previous analysis that the concrete actions of the government and the performance (proxied by the number of deaths) are not predictive for whether people perceive the reactions as too extreme. We need to better understand potential factors that lead to such perceptions. To collect further information about people disagreeing with the strength of government reactions, we therefore conducted our own surveys in Germany in the time periods April 21–23, 2020 and (mainly) May 18–25, 2020, as described in Sect. 3. After removing subjects who did not answer the survey completely, we retained 197 answers in the first, and 248 answers in the second survey which we analyzed further.^5^

We then correlate the variables *perceived too extreme reaction* and *perceived insufficient reaction* with conspiracy tendency (defined as the average answer to all conspiracy-related items) as well as to all single conspiracy items. Some of these items are “directional”, i.e. they strongly suggest that the reaction is too much (e.g., the Bill Gates conspiracy theory). Others are “neutral” in that they have no visible relation to how much a government should react (e.g., it does not seem to matter much whether the virus was produced in a bio lab for the current actions against its spread). If we find a significant correlation for such items, it tells us something about psychological characteristics of the persons in the respective category. Finally, some are a priori “symmetric” in that they could be expected to be higher for both groups of people (e.g., the belief that the media systematically hides information).

The Pearson correlation results are presented in Table 4. We see that there is a strong asymmetry between the two variables: while reactions perceived as “too little” are barely correlated with any conspiracy theory, and for none significantly in both surveys, *perceived too-extreme reaction* is correlated significantly for nearly all of the items in both surveys. The overall score *conspiracies total* is also strongly correlated with the perception of “too extreme” reaction (correlation coefficient above 30% and $p<0.001$), but not significantly with “too little” reaction.

**Table 4:** Correlations of perceived insufficient/too extreme government reaction with various conspiracy-related items and proportion of subjects having heard about them before. The results of first and second survey are printed one below the other

	Perceived reactions		Heard
	Insufficient	Too extreme	About it
The media want to hide information about the coronavirus from us.	.144*(0.043)	.343**(<0.001)	–
	.108 (0.09)	.222**(<0.001)	
The hype about corona was only caused by pharmaceutical companies and other groups that benefit from it.	.065(0.361)	.344**(<0.001)	–
	.088(0.166)	.181**(0.004)	
The virus serves our politicians only as pretext to undermine our basic rights.	.064(0.374)	.399(<0.001)	–
	.214**(0.001)	.232**(<0.001)	
The US secret service developed the virus and brought it to Wuhan in order to specifically damage China.	.061(0.396)	.043(0.550)	49%
	.073(0.254)	.204**(0.01)	51%
China developed the virus in a laboratory for bio-weapons, from where it spread by accident.	− .008(0.916)	.198**(0.005)	70%
	.01(0.876)	.215**(0.01)	75%
Covid-19 is connected to the expansion of the 5G mobile phone network.	.068(0.345)	.092(0.197)	49%
	.141*(0.027)	.047(0.461)	53%
Pharmaceutical companies in conjunction with Bill Gates started the infection in order to make money with a vaccine they had patented.	.019(0.786)	.176*(0.014)	43%
	− .01(0.878)	.950**(<0.001)	75%
Conspiracies total	.078(0.279)	.307**(<0.001)	
	.114(0.074)	.315**(<0.001)	

It is particularly interesting that a perception of too much reaction is highly correlated with items that are “neutral” towards the needed reactions. Compare, e.g., the Bill Gates conspiracy with the Chinese bio weapon conspiracy: while it seems natural that a believer in the former theory might think that COVID-19 is basically just made up and the government is therefore overreacting, there is no such “logical” connection for the second item (bio weapon conspiracy). The most likely explanation for the positive correlation is therefore that a general tendency to believe in conspiracy theories is aligned with a perception of too much government reaction. That such general tendencies exist, i.e., that beliefs in very different and even contradictory conspiracy theories correlate, is a well-known phenomenon (Goertzel 1994).

We need to emphasize here that these results hold for Germany. They probably can be generalized to some extent, but we have seen that in some countries, the number of people complaining about too much reaction from their government is very high. In this case, it is more likely that the problem is with the political orientations rather than with misperception of people having conspiracy tendency. We do not claim that all critics of strict government reactions in Germany follow conspiracy theories, but we do see *on average* a strong relation in our data, at least at the time of the survey. Our results are also in line with the previous finding that conspiracy theories beliefs lower government trust during the COVID-19 pandemic (Karić and Medjedović 2021).

We did a number of robustness tests for our findings: First, we conducted t-tests between the group of persons that perceived a too-strong government reaction and the rest. Again, we obtained a highly significant difference in conspiracies total (12.6 versus 9.6, $p<0.001$, in the first survey; 11.6 versus 9.1, $p<0.001$, in the second survey). For too little government reaction, we did not obtain any significant difference (10.4 versus 9.8, $p=0.33/9.6$ versus 9.3, $p=0.48$).

We also ran linear OLS regressions with dependent variable *perceived too-extreme* or *insufficient reactions* and as independent variables *conspiracies total* together with demographic controls (female, age, student, university degree). For *perceived too extreme reactions*, the only significant variable was *conspiracies total* ($p<0.001$) in both surveys. There is also a significant gender difference in the first survey (with females perceiving the reaction less frequently as too much, $p=0.03$), but this is not significant in the second survey. For *perceived insufficient reactions*, we did not find any significant predictive variables.

We also replaced conspiracy total with conspiracy tendency (a dummy defined as a value of conspiracy total larger than 10), and the regression result did not change decisively: again, there were no significant independent variables for *perceived insufficient reaction*, while for *perceived too-extreme reaction*, the conspiracy tendency was the most significant factor ($p<0.001$), but this time, the coefficient for female failed to be significant.

The connection between conspiracy tendencies and perception of too much reaction can also clearly be seen from Table 5: the proportion of people seeing the reactions as “too strict’ among people without conspiracy tendency is very low (7%/6.9%) as compared to people with conspiracy tendencies (30% / 32.2%). Moreover, Table 6 shows that less than a quarter of the people that do not think the government reacted too strongly have conspiracy tendencies while for the others, the majority has. Conspiracy theories therefore seem to play an important role in forming the perception of too much government reaction, at least in Germany.

**Table 5:** Distribution of perception of government reactions among people with and without conspiracy tendencies

Perception of reaction	First survey		Second survey	
	Conspiracy tendency		Conspiracy tendency	
	No (%)	Yes (%)	No (%)	Yes (%)
Not too strict	94	70	92	73
Rather too strict	6	26	8	23
Far too strict	1	4	0	4
Sum	100	100	100	100

**Table 6:** Distribution of conspiracy tendency among people with different perceptions of government reactions

	Not too strict (%)	Rather too strict (%)	Far too strict (%)
*First survey*			
No conspiracy tendency	79	36	33
Conspiracy tendency	22	64	67
Sum	100	100	100
*Second survey*			
No conspiracy tendency	80	51	0
Conspiracy tendency	20	49	100
Sum	100	100	100

To sum up, the perception of too-extreme government reaction (at least in Germany) seems to be mostly shared by persons who are receptive to conspiracy theories, whereas this is not the case for the opposite opinion of too little reaction.

Finally, we want to add a small analysis on the relation between personal political opinion and political interest on the one hand and belief in conspiracy theories on the other hand. To this end, we use more recent data from new survey waves that elicited such variables (see Sect. 3 for details). We find, as expected, a substantial relation between proxies for a political right-wing attitude (using the aforementioned two variables of the likelihood to vote for the AfD, a right-wing populist party and of seeing the German government as rather right or left): in both cases, the correlation coefficients (Pearson coefficient 0.38, $N=124$, and $-\,0.18$, $N=1617$, respectively) are significant on the 0.1% level. We also find that high political interest correlates with a lower degree of conspiracy beliefs (Pearson coefficient $-0.08$, $N=834$, $p=0.02$).

Table 1 summarises the empirical results. As we expected, the unsatisfactory evaluation of policy responses, either as too extreme or insufficient, is negatively correlated with trust in government to handle the crisis. Media freedom increases the likelihood to judge the government reactions negatively, and reduce the government trust as well. Higher education is associated with higher trust in government. We do find distinct predictive power of education and conspiracy beliefs in assessment of policy responses. Lower-educated people and conspiracy believers tend to perceive government responses as too extreme, but these two aspects are not related to perception of insufficient government reactions. We also higher government effectiveness is associated with higher government trust. The responses by more effective governments are more likely to be judged as too extreme, and less likely to be judged as insufficient. We also find different predictive powers of current and average stringency: the judgment of too-extreme responses are related to average stringency level over time, but not the current stringency level, whereas the judgment of insufficient responses are correlated with current stringency level, but not the average. It seems that people criticises the policy as too extreme tend to be based on general impression of stringency over time, whereas people who perceive insufficient reactions are more focused on stringency level at the time when they fill in the questionnaires.

## Conclusions

Studies show that higher political trust is associated with more compliance in general (van Deth 2017), and higher effectiveness of stringency policies in the case of COVID-19 (Chen et al. 2020). Our study provides an overview of the perception of government actions during the COVID-19 pandemic in March and April 2020 around the world. The results show a large heterogeneity between countries. We find that, on average, stronger and faster stringency of anti-pandemic measures and a smaller number of deaths are positively associated with trust in the governments’ handling of the pandemic. We also find that freedom of the press is negatively associated with this trust.

The most important factor affecting trust in government actions is, however, whether people perceive them as either too little or too much. While the perception of “too little” reaction is partially grounded in factual parameters (amount of objectively measured stringency and the number of COVID-19 deaths as outcome measure), this is not true for the opposite perception of “too much”, which seems to be more driven by sentiments and is more widespread among less educated participants. At least in Germany, this perception is also mainly shared by people who have a tendency towards belief in conspiracy theories.

The results of our paper concern a time of the first big wave of infections around the world. It is possible that the perceptions of people will change in later stages of the pandemic and it will, of course, be very interesting to follow this development. More studies are needed to investigate the extent to which this result can be generalised to different crisis scenarios, as well as to address the causal mechanisms with further data collection and other research methods, such as well-designed experiments and appropriate instrumental variables.

Our study contributes to understanding the determinants of political trust in crisis management. We hope that the current article provides a foundation for further studies on this issue. We also hope that it can provide valuable insights into people’s reactions to government actions that could be useful in similar situations in the future. One short message from our study to policy makers worldwide is that, in the case of the COVID-19 pandemic, government unresponsiveness is more likely to cause dissatisfaction and distrust from the public than too much response. Since the public who are dissatisfied with insufficient responses to COVID-19 tend to be more silent than the public who perceive actions as too extreme, the silent majority may be invisible to policy makers in democratic societies, which leads to policies oriented towards too loose/slow reaction in those societies, as we see has happened in the COVID-19 pandemic.

Are conspiracy beliefs merely a reflection of a lack of ability to process information? There is an emerging literature on motivated beliefs that suggest misbeliefs are not necessarily caused by lack of cognitive ability but rather motivated by ideology (Kahan 2013). Therefore, the tendency to believe in conspiracies can be influenced by political orientations. Our survey also investigated further the relationship between conspiracy theory beliefs and political orientations. We find that a higher tendency towards conspiracy beliefs correlates to right-wing attitudes and lower interest in politics.

One limitation is that our data did not distinguish trust and distrust. Therefore, it is important to understand which factors potentially determine political trust. Van De Walle and Six (2014) argued that trust and distrust are distinct concepts: low trust is not equal to high distrust, and low distrust is also not equivalent to high trust. In the future, it would help us to gain more insight to treat trust and distrust as two distinct concepts.

## Footnotes

1 Data from Rieger and He-Ulbricht (2020) on political opinions includes more recent survey waves than the ones described in the main part of our paper, the latest one from January 2021.

2 We evaluate both surveys separately, since in this highly dynamic situation, effects may potentially differ over time.

3 The wording differs slightly, since the survey questions were not copied, but independently designed. That they are basically the same is a fortunate coincidence for our analysis.

4 A priori, it could be possible that the relation between government trust and stringency measures is just an artifact of a different relation, namely that governments in countries where people have a high confidence in politicians and government can more easily enforce stringency measures and in such countries, people will also trust the government more to handle the crisis, simply because they *always* trust the government more. We therefore used data from the World Value Survey on country average confidence in the government and added it to the above regressions, but the variable was never statistically significant and, in most models, did not change the significance levels of the relevant variables. It seems, therefore, that trust in the government handling the pandemic well is directly related to stringency measures and not only indirectly via a previously existing confidence in the government.

5 There were no statistically significant differences regarding age, gender, student status or university degree between those who completed the survey and those who dropped out after answering the first page of the survey (which contained the demographic questions).
